# Kallikrein-related peptidase 6 induces chemotherapeutic resistance by attenuating auranofin-induced cell death through activation of autophagy in gastric cancer

**DOI:** 10.18632/oncotarget.13352

**Published:** 2016-11-15

**Authors:** Tae Woo Kim, Seon-Jin Lee, Jong-Tae Kim, Sun Jung Kim, Jeong-Ki Min, Kwang-Hee Bae, Haiyoung Jung, Bo-Yeon Kim, Jong-Seok Lim, Young Yang, Do-Young Yoon, Yong-Kyung Choe, Hee Gu Lee

**Affiliations:** ^1^ Immunotherapy Convergence Research Center, Korea Research Institute of Bioscience and Biotechnology, Daejeon, Republic of Korea; ^2^ Department of Biomolecular Science, University of Science and Technology (UST), Daejeon, Korea; ^3^ Department of Life Science, Dongguk University-Seoul, Seoul, Republic of Korea; ^4^ Biotherapeutics Translational Research Center, Korea Research Institute of Bioscience and Biotechnology, Daejeon, Republic of Korea; ^5^ Research center for metabolic regulation, Korea Research Institute of Bioscience and Biotechnology, Daejeon, Republic of Korea; ^6^ World Class Institute, Korea Research Institute of Bioscience and Biotechnology, Ochang, Cheongwon, Republic of Korea; ^7^ Department of Biological Sciences, Sookmyung Women's University, Seoul, Republic of Korea; ^8^ Department of Bioscience and Biotechnology, Konkuk University, Seoul, Republic of Korea

**Keywords:** kallikrein-related peptidase 6, autophagy, auranofin, cell death, chemoresistance

## Abstract

Kallikrein-related peptidase 6 (KLK6) is a biomarker of gastric cancer associated with poor prognosis. Mechanisms by which KLK6 could be exploited for chemotherapeutic use are unclear. We evaluated auranofin (AF), a compound with cytotoxic effects, in KLK6-deficient cells, and we investigated whether KLK6 expression induces autophagy and acquisition of drug resistance in gastric cancer. Using cultured human cells and a mouse xenograft model, we investigated how KLK6 affects antitumor-reagent-induced cell death and autophagy. Expression levels of KLK6, p53, and autophagy marker LC3B were determined in gastric cancer tissues. We analyzed the effects of knockdown/overexpression of KLK6, LC3B, and p53 on AF-induced cell death in cancer cells. Increased KLK6 expression in human gastric cancer tissues and cells inhibited AF-induced cell motility due to increased autophagy and p53 levels. p53 dependent induction of KLK6 expression increased autophagy and drug resistance, whereas *KLK6* silencing decreased the autophagy level and increased drug sensitivity. During AF-induced cell death, KLK6 and LC3B colocalized to autophagosomes, associated with p53, and were then trafficked to the cytosol. In the xenograft model of gastric cancer, KLK6 expression decreased AF-induced cell death and KLK6-induced autophagy increased AF resistance. Taken together, the data suggest that the induction of autophagic processes through KLK6 expression may increase acquisition of resistance to AF. Our findings may contribute to a new paradigm for tumor therapeutics.

## INTRODUCTION

Gastric cancer is widespread and among the most lethal of cancers [1]. Advancements in biomedical science have led to improved therapies and diagnostics [2]; however, the problem of drug resistance remains a major obstacle to successful therapeutic approaches against the progression of gastric cancer and indicates a critical need for novel therapeutic approaches [3]. Increased expression of drug pumps or detoxification enzymes [4] and induction of autophagy contribute to treatment failure [5]. Recently, combined chemotherapy has been found to be a superior treatment strategy [6]. Hence, the search for effective chemosensitizers that can augment the efficiency of anticancer drugs and circumvent multi-drug resistance has increased [7, 8]. Multiple reports have suggested that autophagy is related to anticancer activity and apoptosis of cancer cells, thus providing an attractive strategy for improving anticancer therapy [9].

The regulation and function of autophagy in human disease is currently not well understood. Recent reports have identified autophagy functions associated with cancer cell survival or death, and the concept that tumor cells have evolved through genetic and epigenetic changes to require autophagy under basal conditions has been proposed [10]. Autophagy is a self-cannibalization process in which bulk cytosolic components are sequestered in the autophagosome for lysosomal degradation [11]. Owing to its basic role in lysosomal degradation and recycling of metabolic precursors, autophagy is generally considered a cell survival pathway [12]. However, autophagy plays a dual role in cancer, contributing to both suppression of cancer initiation and promotion of cancer growth [13]; thus, control of its function would be an important therapeutic strategy [14]. Autophagy is often activated as a survival pathway in chemotherapy-treated tumor cells but becomes a death signal after several treatments. Several reports have shown that apoptosis regulators can control autophagy and that autophagy also contributes to apoptosis in biological process such as tissue homeostasis, development, and disease [15]. Autophagy is regulated by oncogenes and tumor suppressors [16] such as p53 [17]. The induction of autophagy by p53 activation is regulated by transcription-dependent and independent mechanisms [18]. In contrast to these positive effects on autophagy, it has been shown that depletion of p53 function is sufficient to trigger full activation of autophagy [19]. Moreover, autophagy is activated by nuclear, and suppressed by cytoplasmic p53 [20].

Auranofin (AF) has been suggested to act as an inhibitor of thioredoxin reductase, leading to modifications of the cellular redox status resulting in overproduction of reactive oxygen species (ROS), oxidative damage, and apoptosis [21, 22]. AF exerts a strong cytotoxic effect on several types of neoplastic cells both *in vitro* and *in vivo* [23, 24]. Moreover, study of the effects of AF in gastric cancer revealed that AF overcame apoptosis resistance mediated by an anti-cancer drug [25], suggesting that AF may have potential for tumor chemotherapy for various tumors as well. Accordingly, the use of AF to treat various cancers has been explored [25, 26], and AF is currently in clinical trials for the treatment of leukemia [27]. However, the usability and action of AF in gastric cancer have not yet been demonstrated. These findings suggest that repositioning drugs for AF may be a promising approach for cancer treatment.

We previously reported that the serine protease kallikrein-related peptidase 6 (KLK6) is a potential biomarker for colon and gastric cancer because it is highly expressed in these cancers and is important in tumorigenesis [28]. Recent reports of an association between elevated KLK6 expression in primary ovarian tumors and poor prognosis indicate that KLK6-positive patients have increased risk of relapse and death [29]. KLK6 overexpression confers chemoresistance to paclitaxel and enhances cell survival via integrins which is regulated by cell adhesion as contributors to chemoresistance and metastatic progression [30, 31]. Here, KLK6 may be an autophagy-related and p53-dependent gene in several tumor microenvironments. Our results suggest that modulation of KLK6 status to regulate AF-induced autophagic cell death is a potential therapeutic strategy for gastric cancer. We demonstrate that KLK6 overexpression via induction of autophagy may contribute to acquired chemoresistance in gastric cancer.

## RESULTS

### KLK6 expression increases stage-dependently in gastric cancer and is related with resistance to AF-induced cell death

**Figure 1:**
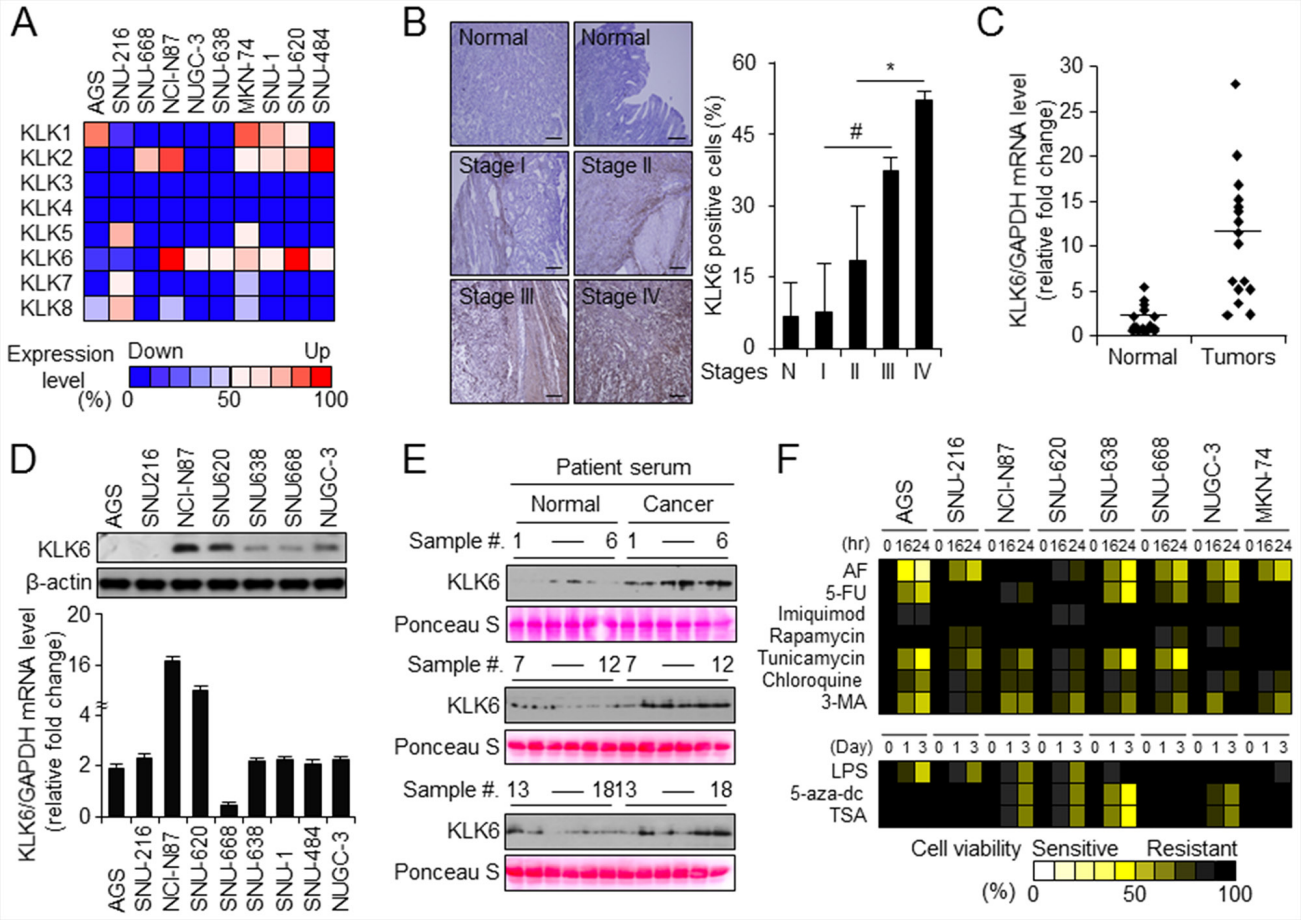
KLK6 expression is upregulated *in vivo* and *in vitro* in late-stage gastric cancer **A**. RT-PCR analysis of KLK1–8 expression compared relative intensity with GAPDH expression in the indicated gastric cancer cell lines. The intensity of each KLK1-7 mRNA band was quantified and normalized with that of the corresponding GAPDH band (ATTO Densitograph software library). **B**. Representative immunohistochemical images (left) and microarray-based quantitation (right) of KLK6 expression in normal (n = 59) and gastric cancer tissues at indicated stages (stage I, n = 8; II, n = 14; III, n = 24; and IV, n = 3). Original magnification, 200×; scale bars, 50 μm; *P < 0.05. **C**. KLK6 expression in 16 pairs of normal and cancer tissues from gastric cancer patients normalized to the GAPDH expression level as determined with qPCR. **D**. Western blot (top) and quantitation of real-time RT-PCR (bottom) data of KLK6 expression in the indicated cell lines. **E**. Western blot analysis of KLK6 levels in serum of gastric cancer patients and normal controls (n = 18 pairs) normalized to Ponceau S staining intensities. **F**. Cell viability of gastric cancer cells treated with AF (2.5 μM), 5-FU (2.5 μM), imiquimod (2.5 μM), rapamycin (10 nM), tunicamycin (2.5 μM), chloroquine (20 μM), 3-MA (5 mM), LPS (1 μM), 5-aza-dc (1 μM), and TSA (100 nM) for the indicated times as measured by WST-1 assay. The viability of drug-treated cells was expressed relative to that of DMSO-treated control cells, whose viability was set at 100%.

We examined the levels of *KLK1–8* mRNAs compared with *GAPDH* mRNA in various gastric cancer cell lines using RT-PCR (Figure 1A). In several gastric cancer cell lines (AGS, SNU-216, SNU668, NCI-N87, NUGC-3, SNU-638, MKN-74, SNU-1, SNU-620, and SNU-484), *KLK6* expression was higher than that of other KLK family members. Immunohistochemistry (IHC) revealed higher KLK6 expression in gastric cancer tissues than in paired normal gastric tissues, and expression was tumor-stage-dependent (Figure 1B). KLK6 mRNA levels in lung, pancreas, liver, breast, and colon tissues and KLK6 mRNA and protein levels in various gastric cancer cell lines indicated different patterns of KLK6 expression (Supplementary Figure S1A–S1C). Especially, we investigated KLK6 mRNA and protein levels using qPCR and western blot analysis in normal and gastric tumor tissues, and in gastric tumor cell lines such as AGS, SNU-216, NCI-N87, SNU-620, SNU-668, SNU-638, SNU-1, SNU-484, and NUGC-3 (Figure 1C and 1D). KLK6 mRNA was approximately 6-fold higher in cancer tissues than in normal tissues and in NCI-N87 and SNU-620 cells than in the other cell lines. Moreover, KLK6 levels were approximately 5-fold higher in gastric cancer patient sera than in normal sera (Figure 1E). Treatment with secreted KLK6 protein did not markedly increase cell proliferation but dose-dependently increased the autophagy level in AGS and SNU-216 cells (Supplementary Figure S1D and S1E).

Most anticancer drugs are currently focused on the drug repositioning for overcoming chemotherapy resistance [32]. To investigate the relationship between drug-induced cell death and KLK6 expression, we performed a cell viability assay upon treatment with various drugs in the gastric cancer cell lines. For most drugs, the cytotoxic effect was not related to the KLK6 expression level; however, the cytotoxicity of the anti-leukemia drug AF was negatively related with KLK6 mRNA and protein levels (Figure 1F); i.e., KLK6 expression was higher in AF-resistant NCI-N87 and SNU-620 than in AF-sensitive AGS and SNU-216 cells (Figure 1D and 1F). In summary, the KLK6 expression level was higher in gastric cancer tissue and cell lines such as AGS, SNU-216, NCI-N87, SNU-620, SNU-638, SNU-668, NUGC-3, and MKN-74 than in normal cells, and AF-resistant gastric cancer cells (NCI-N87 and SNU-620) exhibited the highest levels of KLK6.

### Gastric cancer cell resistance to AF depends on the KLK6 expression level and autophagy

**Figure 2:**
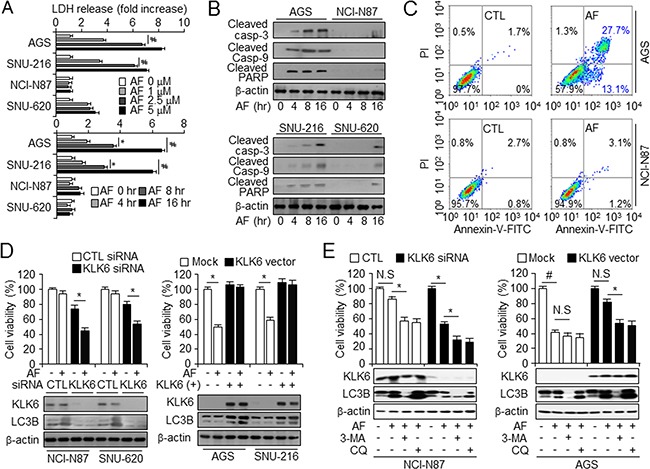
KLK6 expression is attenuated by AF-induced cell death in gastric cancer cell lines **A**. LDH release from NCI-N87 and SNU-620 (AF-induced chemoresistant cell lines) and AGS and SNU-216 (AF-induced chemosensitive cell lines) cells incubated with DMSO or the indicated concentrations of AF (top). Cells treated with AF (2.5 μM) were further incubated for the indicated times (bottom). Values are the mean ± SD from triplicate experiments; *P < 0.05, #*P* < 0.01, **B**. Western blot analysis of cleaved caspase-3, caspase-9, and PARP in the indicated cell types treated with AF (2.5 μM) for the indicated times. **C**. FACS analysis of annexin V and PI staining in AGS (top) and NCI-N87 (bottom) cells treated with DMSO (CTL) or AF (2.5 μM). **D**. After transfection with control (CTL) or KLK6 siRNA (left panel) and KLK6 overexpression or mock vector (right panel) in the presence and absence of AF (2.5 μM) treatment, cell viability and expression of KLK6 and LC3B in AGS, SNU-216, NCI-N87, and SNU-620 cells were measured by WST-1 assay and western blot analysis, respectively. **E**. Cell viability assay and western blot analysis of KLK6, and LC3B in NCI-N87 and AGS cells following transfection with control (CTL) or KLK6 siRNA (left panels) and KLK6 overexpression plasmid or mock vector (right panels) in the presence and absence of AF (2.5 μM) and 3-MA (5 mM) or CQ (20 μM), *P < 0.05.

Because high KLK6 expression seemed to correlate with AF chemotherapy resistance *in vitro*, we further investigated the relationship between KLK6 expression and cell viability in AF-treated cells using WST-1 viability and annexin-V PI staining assays, qPCR, and nuclear fragmentation (Supplementary Figure S2A–S2E). All cell lines, with the exception of NCI-N87 and SNU-620, showed significantly increased AF-induced cell mortality at concentrations from 2.5 μM in a dose-dependent manner. Lactate dehydrogenase (LDH) release, an indicator of cell lysis, qPCR, and WST-1 assay showed that cells with high KLK6 mRNA and protein expression levels showed dose- and time-dependent resistance to AF when compared to cells with lower KLK6 expression (Figure 2A and Supplementary Figure S2A-S2C). Reduced viability of the AF-sensitive cells AGS and SNU-216 was due to induction of apoptotic events, including cleavage of caspase-3, caspase-9, and PARP (Figure 2B). After AF treatment, AF-resistant cells contained few annexin V, PI-positive and DAPI-positive cells, whereas AF-sensitive cells showed an increase in annexin V and PI-positive cells (Figure 2C and Supplementary Figure S2D and S2E). siRNA-mediated knockdown of KLK6 in the AF-resistant NCI-N87 and SNU-620 cells lowered the cell viability, and reduced the expression of the autophagy marker LC3B (Figure 2D, left). Conversely, KLK6 overexpression increased the cell viability and LC3B expression in AF-sensitive AGS and SNU-216 cells (Figure 2D, right). These experiments indicated that KLK6 overexpression inhibits AF-induced cell death and that the low level of autophagy might be associated with AF-induced chemotherapy resistance.

To confirm the involvement of autophagic cell death in acquired resistance to chemotherapy under AF treatment, we inhibited autophagy using 3-MA and CQ in KLK6 overexpression and knockdown cells. The overexpression of KLK6 in KLK6-negative AGS cells increased AF-induced chemotherapy resistance as compared with the mock. Blockage of autophagy flux by 3-MA and CQ was verified by western blot analysis of KLK6, and LC3B (Figure 2E). After *KLK6* silencing in KLK6-positive NCI-N87 cells, we observed increased cell mortality and attenuated autophagy upon AF treatment in the presence of autophagy inhibitors, (Figure 2E, left panel). 3-MA and CQ treatment decreased the activation of autophagy and reversed AF-mediated cell death and thus had a chemosensitizing effect. KLK6 overexpression promoted KLK6 expression and autophagy flux by LC3B activation to a greater extent in KLK6-transfected than in mock-transfected AGS cells (Figure 2E, right panel). These findings demonstrated a link between KLK6 expression and autophagic cell death that might give a clue for tumor promotion and resistance to targeted therapy.

### Increased KLK6-induced autophagy attenuates AF-induced gastric cancer cell death

**Figure 3:**
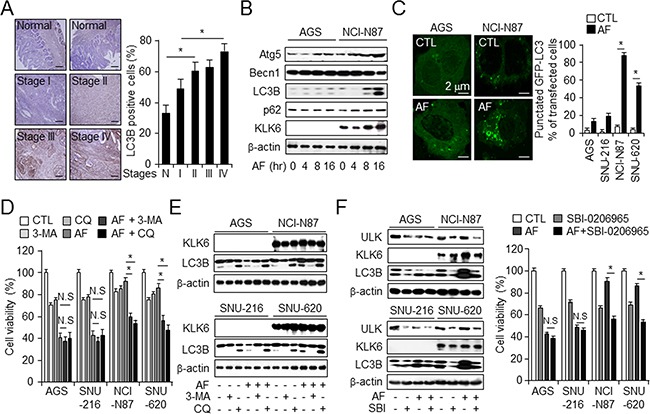
KLK6 expression decreases in AF-induced cell death *via* the activation of autophagy in gastric cancer **A**. Representative immunohistochemical images and microarray-based quantitation of LC3B expression in normal and cancer tissues at the indicated stages used in Figure 1B. Original magnification, 200×; scale bars, 50 μm; *P < 0.05. **B**. Western blot analysis of expression of indicated autophagy markers in indicated cell types treated with AF (2.5 μM) for the indicated times. **C**. Fluorescence microscopic analysis and quantitation of autophagy activation, characterized by punctate LC3B staining, in indicated cell types transfected with pEGFP-LC3B and treated with AF (2.5 μM) or DMSO for 16 h. *P < 0.05. Cell viability **D**. and western blot analysis of KLK6 and LC3B **E**. in the indicated cell types treated with DMSO or a combination of AF and autophagy inhibitor 3-MA (5 mM) or CQ (20 μM) for 16 h. **F**. Western blot analysis of KLK6 and LC3B expression (left) and cell viability (right) in the indicated cell types treated with AF (2.5 μM) in the presence or absence of SBI-0206965 (SBI) (10 μM) for 16 hr; * *P* <0.05.

Autophagy induction is important for drug resistance in various cancer environments [33]. A recent report showed that LC3B is a potential prognostic marker in breast cancer [34]. We hypothesized that LC3B may serve a similar function in gastric cancer. We examined basal autophagy levels based on LC3B expression in the normal and tumor tissues such as lung, pancreas, liver, breast, colon and lung, liver, breast, and gastric cancer cell lines (Supplementary Figure S3A–S2C) and LC3B levels in patients with different stages of gastric cancer (Figure 3A). LC3B was expressed at ~15% higher level in stage I tumors than in normal tissue, but it was highly expressed (25–40%) in stage II, III, and IV tumors. Autophagy markers such as LC3B and Atg5, and KLK6 which is gastric cancer biomarker, but not beclin-1, showed increased expression over 16 h after AF treatment (Figure 3B and Supplementary Figure S3D). Next, we examined the presence of autophagosomes using pEGFP-LC3B vector and cyto-ID autophagy assay, and monodansyl-cadaverine (MDC) staining (Figure 3C and Supplementary Figure S3E–S3G). Increased punctated LC3B, cyto-ID, and MDC-positive cells were detected in AF-resistant cells NCI-N87 and SNU-620, corroborating that high-level KLK6 expression is related with increased autophagy in AF-treated cells. To confirm that the LC3B expression pattern reflected autophagic processes, we investigated whether autophagy inhibitors increased AF-induced cell death in both of high and low KLK6 expression cell lines. Treatment of NCI-N87 and SNU-620 cells with 3-MA and chloroquine (CQ) significantly enhanced AF-induced cell death and inhibited autophagic flux, while it did not affect these parameters or LC3B punctae in AF-sensitive AGS and SNU-216 cells (Figure 3D and 3E). This result further supports an important role for KLK6 in the regulation of AF-induced autophagic cell death and suggests that combined treatment with AF and autophagy inhibitor might increase chemotherapeutic effects in drug-resistant gastric cancer cells.

To determine whether increases in KLK6 expression correlated with autophagic flux during AF treatment, we repeated these experiments, treating cells with AF in the presence or absence of SBI-0206965 (SBI) as ULK1 inhibitor. SBI treatment further reduced LC3B-II form in high-KLK6 cell lines under DMSO and AF treatments, indicative of autophagic activity (Figure 3F, left). To confirm that the LC3B expression pattern reflects the autophagic processes, we used a WST-1 assay to determine whether the autophagy inhibitors affected AF-induced cell viability in high- and low-CST1 cell lines. Treatment of high-KLK6 cell lines (NCI-N87 and SNU-620) with SBI significantly enhanced AF-induced cell death, while the presence of the autophagy inhibitors did not affect the mortality rate of AF-treated low-KLK6 cell lines (AGS and SNU-216) (Figure 3F, right). Together, these findings suggested a crosstalk between KLK6 and LC3B and indicate that autophagy might protect against AF-induced cell death.

### p53 is important for upregulation of KLK6 and autophagic induction

**Figure 4:**
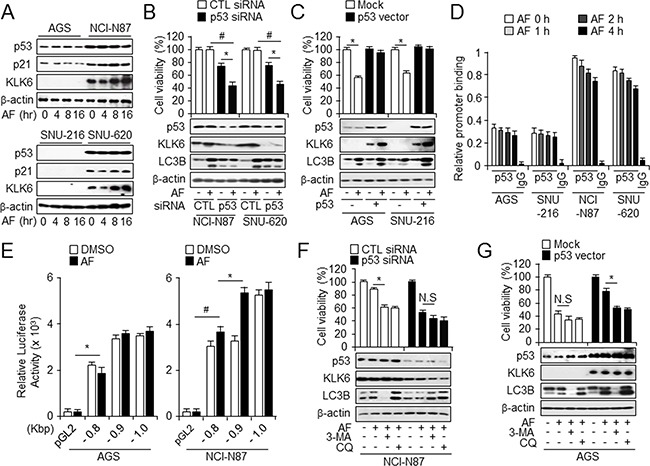
Enrichment of p53 is important for AF-induced KLK6 expression and autophagic activation **A**. Western blot analysis of KLK6, p53, and p21 levels in AF-induced AGS and NCI-N87 cells. **B**. After transfection with control or p53 siRNA in the presence or absence of AF (2.5 μM) treatment, cell mortality and expression of p53, KLK6, and LC3B were measured by WST-1 assay and western blot analysis in NCI-N87 and SNU-620 cells. **C**. After transfection with p53 overexpression or mock vector in the presence or absence of AF treatment, cell viability and the expression level of the indicated proteins in AGS and SNU-216 cells were measured by WST-1 and immunoblot assay. **D**. Chromatin immunoprecipitation (ChIP) analysis of relative *KLK6* promoter binding to p53 in the indicated cell types treated with AF for the indicated times. IgG was included as a negative control. **E**. The 1-kb sequence upstream of *KLK6* was divided into the three indicated regions that were cloned into luciferase reporter vector pGL3. After transient transfection into AGS and NCI-N87 cells, luciferase activity was measured using a triple luciferase assay. *P<0.05. After transfection of KLK6 siRNA or control siRNA **F**. in NCI-N87 (KLK6-positive) cells and p53 overexpression plasmid or mock vector **G**. into AGS (KLK6-negative) cells, cells were treated with AF alone, AF plus 3-MA, or AF plus CQ. For measuring the level of autophagy flux, cell viability was detected using WST-1 assay and expression of indicated proteins by immunoblot assay using KLK6, LC3B and p53 antibodies. *P < 0.05.

IFN-γ-induced autophagy is increased in p53^+/+^, but reduced in p53^-/-^ HCT116 colon cancer cells, suggesting that IFN-γ-induced autophagy is p53-dependent [35]. One of the best-known p53 target genes is p21, which regulates cell survival [36] and protects cells against apoptosis [37]. Western blot analysis showed a time-dependent expression of the KLK6, p21, and p53 levels after AF treatment in NCI-N87 and SNU-620 cells when compared to AF-sensitive cells, suggesting a role for these proteins in AF-induced cell death (Figure 4A). The levels of p21 and p53 did not change in time-dependent manner after AF treatment in NCI-N87 and SNU-620 cells, although they are higher than in control. We hypothesized that increased KLK6 and p53 expression contributed to acquired resistance to AF-mediated cell death and that augmented apoptotic events in AF-sensitive cells likely reflect low KLK6 and p53 levels. To determine whether loss/gain of p53 function influenced AF-induced autophagic cell death, p53 expression in tissues of gastric tumor patients was analyzed by IHC. p53 expression was higher in tumor than in normal tissues and associated with high-grade tumors, similar to the KLK6 expression pattern (Supplementary Figure S4A and S4B). Additionally, p53 was upregulated in KLK6-overexpressing NCI-N87 and SNU-620 cells as compared to SNU-216 cells (Supplementary Figure S4C). NCI-N87 and SNU-620 cells, with high levels of KLK6 and p53, were transfected with control or p53 siRNA and treated with AF. In AF-resistant p53 knockdown cells, AF treatment decreased cell survival and KLK6 and p53 levels, and attenuated autophagy activation (Figure 4B). Conversely, AF-sensitive AGS and SNU-216 cells overexpressing p53 showed increased KLK6 and p53 levels and no difference in viability after AF treatment (Figure 4C). Next, genetic analysis of the promoter region of *KLK6* revealed consensus binding sites for the p53 transcription factor using chromatin immunoprecipitation (ChIP). We hypothesized that p53 might increase *KLK6* expression and autophagy during AF treatment. Chromatin immunoprecipitation assays revealed that p53 was rapidly induced to bind the *KLK6* promoter after stimulation by AF treatment in AF-resistant, but not AF-sensitive cells (Figure 4D). To confirm the role of *KLK6* promoter activity in AF-treated KLK6^high^ and KLK6^low^ cells, we measured promoter activity within the region -902 to -13 upstream of *KLK6* using a luciferase reporter vector in AF-treated cells. Only the promoter containing the region -902 to -802 had higher luciferase-reporter activity in AF-treated cells than in DSMO-treated cells, and this effect was observed only in AF-resistant NCI-N87 cells (Figure 4E). p53 binding to the *KLK6* promoter may be involved in the regulation of KLK6 expression and AF-induced cell death. To further clarify the roles of KLK6 and p53 in autophagy induction, we assayed autophagy flux after treatment with 3-MA and CQ. Because AF-induced cell death was specifically regulated in accordance with KLK6 and p53 status, we hypothesized that either blockage of early- or late-phase autophagy or KLK6 and p53 silencing would increase susceptibility to AF-mediated cell death. To determine the effect of p53 status on AF-induced cell death, we overexpressed/knocked down p53 and used 3-MA and CQ to increases lysosomal pH that prevents autophagosome -lysosome fusion as well as degradation. After p53 overexpression in AGS cells weakly expressing p53, AF-induced cell death decreased with 3-MA or CQ treatment, and p53 overexpression also increased the KLK6 and autophagy levels. These results indicated that KLK6 expression increased autophagy and attenuated AF-induced autophagic cell death through p53 (Figure 4F). In contrast, after p53 knockdown in NCI-N87 cells, which highly express p53, cell survival decreased most strongly after cotreatment with AF and 3-MA or CQ, which also downregulated the p53 and KLK6 levels compared to cells transfected with control siRNA. Moreover, LC3B expression was downregulated by autophagy inhibitors in the presence of low p53 and KLK6 levels (Figure 4G).

### KLK6 expression reduces AF-induced tumor suppression in a xenograft model

**Figure 5:**
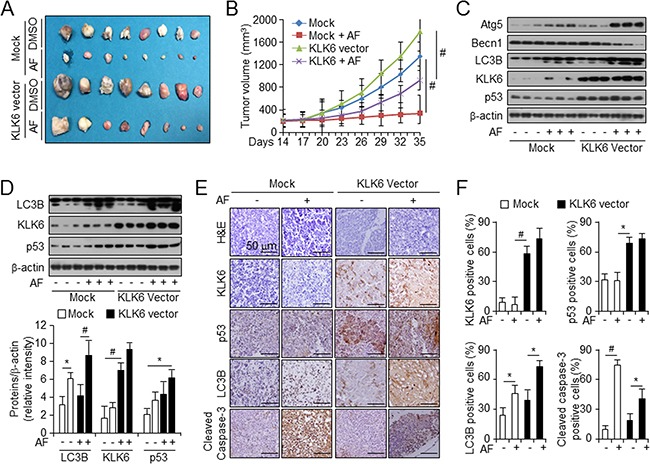
KLK6 overexpression with AF treatment decreases therapeutic effects on xenograft tumor growth *in vivo* **A**. Image of xenograft tumors from mice treated with mock or KLK6 expression vector, with or without AF treatment. **B**. Tumor volumes on the indicated days after injection of cells transfected with indicated vectors in the presence or absence of AF treatment into mice. Values are the mean ± SD for each group (n = 8) **C**. Western blot analysis of the indicated proteins in tumors from cells transfected with the indicated vectors in the presence and absence of AF treatment of mice **D**. Western blot analysis (top) and quantitation (bottom) of the indicated proteins normalized to β-actin in spleens from mice of each treatment group. *P < 0.05; #*P* < 0.01. **E**. Hematoxylin and eosin staining (top row) and immunohistochemical analysis of the indicated proteins (lower rows) of sections of indicated types of xenograft tumors. Original magnification, 200×; scale bars, 50 μm. **F**. Quantification of immunohistochemical staining in (E). *P < 0.05; #*P* < 0.01.

Next, we tested whether our finding that KLK6 expression level is a crucial factor in AF-mediated autophagic cell death *in vitro* could be reproduced *in vivo*. We overexpressed KLK6 in AGS cells and injected mice with a tumor cell load of 1 × 10^7^ cells to form a xenograft. Then, mice were injected intraperitoneally (i.p.) with AF to analyze its effects according to the KLK6 level. Effects of differential KLK6 expression and AF treatment on tumor volume are shown in Figure 5A. Tumors from KLK6-overexpressing cells had larger volumes than those from mock-transfected cells. AF treatment decreased the tumor volume for both mock and KLK6-overexpressing cells (Figure 5B). These results suggested that AF-induced growth inhibition of tumor from mock-transfected cells was associated with an increase in autophagic cell death as compared with tumors in KLK6-overexpressing cells. Consistent with our *in vitro* data, expression of KLK6, p53, ATG5 and LC3B activation in tumor lysates from AF-treated mice with tumors from KLK6-overexpressing cells was higher than that in lysates from mock-transfected cell tumors (Figure 5C). Western blot analysis of protein extracts from the spleen, lungs, and kidneys of mice gave results similar to those obtained with tumor lysates (Figure 5D and Supplementary Figure S5A and S5B). To determine whether differential KLK6 expression affected AF-induced autophagic cell death and autophagy processes, we analyzed tumor sections using H&E staining and IHC for KLK6. We compared the levels of KLK6, p53, LC3B, and cleaved caspase-3 to *in vivo* data (Figure 5E). IHC analysis of tumor sections using anti-cleaved caspase-3 antibody indicated that tumors from mock-transfected AGS cells expressed lower levels of KLK6 and p53 than those from KLK6-overexpressing cells. The expression levels of LC3B, KLK6, and p53 were approximately 2-fold, 4-fold, and 2-fold higher, respectively, in the KLK6-overexpressing group than in the mock-transfected cells (Figure 5F). Together, these findings suggested that AF-induced autophagic cell death may depend on KLK6 and p53 expression *in vivo* as well as *in vitro*.

### p53 and KLK6 localize to nucleus and interact with LC3B

**Figure 6:**
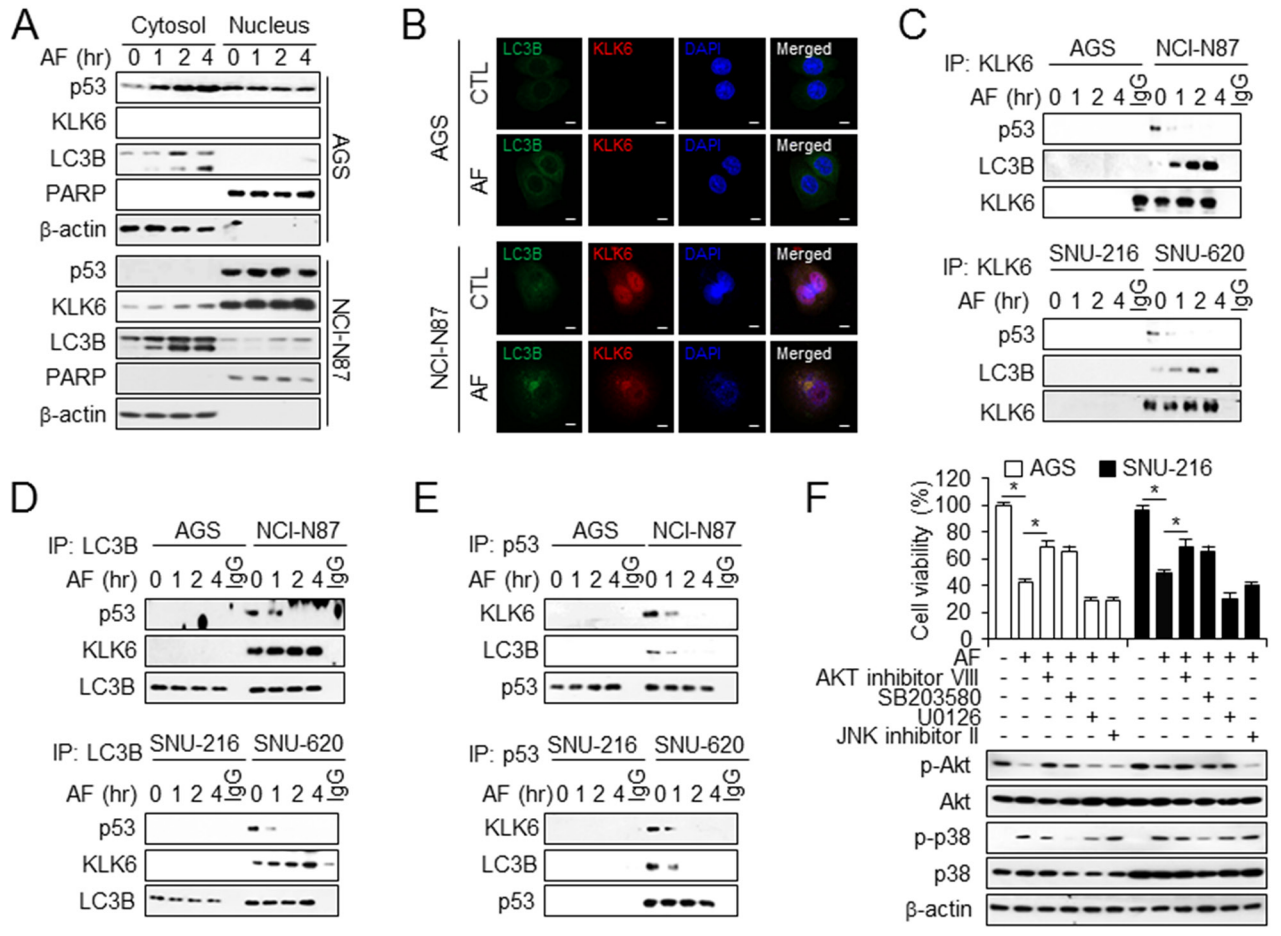
Interaction of KLK6, LC3B, and p53 is required for AF-induced autophagy activation **A**. Western blot analysis of the indicated proteins in cytosolic and nuclear extracts of AGS (top) and NCI-N87 (bottom) cells treated with AF or DMSO for the indicated times. β-actin and PARP served as cytosolic and nuclear loading controls, respectively. **B**. Colocalization of KLK6 and LC3B on gastric cancer cells. AGS and NCI-N87 cells were treated with AF, and stained at 4°C with KLK6 and LC3B antibody. After primary staining, also stained with Alexa488 (green) and Alexa555 (red), and nuclear stained with DAPI. Colocalization is represented by yellow appearance in the merge. Western blot analysis of KLK6, LC3B, and p53 co-immunoprecipitated (IP) with anti-KLK6 **C**. anti-LC3B **D**. and anti-p53 **E**. from AF-induced chemosensitive (AGS and SNU-216) and chemoresistant (NCI-N87 and SNU-620) cells treated with AF for the indicated times using p53, KLK6, and LC3B antibodies. **F**. Viability (top) and Western blot analysis of total and phosphorylated Akt and p38 (bottom) in AGS and SNU-216 cells treated with Akt inhibitor VIII (10 μM), p38 inhibitor SB203580 (30 μM), MEK1, 2 inhibitor U0126 (20 μM), and JNK inhibitor II (20 μM). *P < 0.05.

Cytoplasmic p53 inhibits autophagy by interacting with FIP200, a protein involved in autophagy, whereas nuclear p53 binds the promoter region of pro-autophagic genes to induce autophagy [38]. To determine whether subcellular localization of p53, KLK6, and LC3B correlated with AF-induced cell death, we examined p53, KLK6, and LC3B localization in control and AF-treated cells (Figure 6A). We isolated cytosolic and nuclear fractions of AGS and NCI-N87 cells. In AF-resistant NCI-N87cells, high levels of p53 and KLK6 were found in the nuclear fraction and cytosolic p53 was lowly expressed, while LC3B was predominant in the cytosolic fraction. We further evaluated the nuclear localization of p53 and KLK6 using confocal microscopy. p53, KLK6, and LC3B localized to the cytosolic region diffused cytoplasmic staining under control conditions. AF treatment induced the translocation of both p53 and KLK6 from cytosol to the nucleus in NCI-N87. Immunofluorescence analysis confirmed that KLK6 localized more strongly with LC3B and p53 in AF-induced resistant cells than in AF-sensitive cells. However, p53–LC3B co-localization was not observed (Figure 6B and Supplementary Figure S6A and S6B). To examine whether LC3B is involved in p53-induced KLK6 expression in AF treatment and in trafficking of p53 and KLK6, and whether AF-mediated autophagic cell death involves p53 regulation, cells were exposed to AF and immunoprecipitated fractions were isolated using antibodies. p53 did not interact with either KLK6 or LC3B in AF-sensitive AGS and SNU-216 cells, whereas both KLK6 and LC3B interacted with p53 in AF-resistant NCI-N87 and SNU-620 cells. In DMSO-treated NCI-N87 and SNU-620 cells, an interaction between KLK6 and p53 was detected in immunoprecipitated fractions by co-immunoprecipitation, indicating the localization of KLK6 and p53 in association with LC3B. Treatment of these cells with AF caused a time-dependent disruption of this complex, although the interaction between KLK6 and LC3B was not affected (Figure 6C). An interaction of p53 with LC3B was also detected in autophagosomes from AF-resistant cells under DMSO treatment, with time-dependent dissociation of this complex under AF treatment (Figure 6D). Similar results were obtained by immunoprecipitation using p53 antibody (Figure 6E). In brief, interactions among LC3B, KLK6, and p53 were observed during AF treatment in AF-resistant cells, and KLK6 and p53 are important factors in AF-mediated autophagic cell death.

AF-induced cell death may be associated with a cascade transmitted to caspase-3 and -9 through Akt and p38 induction [39]. Therefore, we investigated the downstream signaling pathway of AF-induced autophagic cell death to determine molecular mechanisms underlying AF function. We tested the effects of Akt inhibitor VIII, p38 inhibitor SB203580, MEK1, 2 inhibitor U0126, and JNK inhibitor II on AF treatment in AF-sensitive AGS and SNU-216 cells. Only VIII and SB203580 protected against AF-induced cell death and enhanced viability (Figure 6F). Western analysis of the same samples demonstrated an increased p-AKT level upon treatment with VIII and inhibition of p38 phosphorylation after SB203580 treatment (Figure 6F).

### Autophagy induction by KLK6-overexpressing cells with chemoresistance

**Figure 7:**
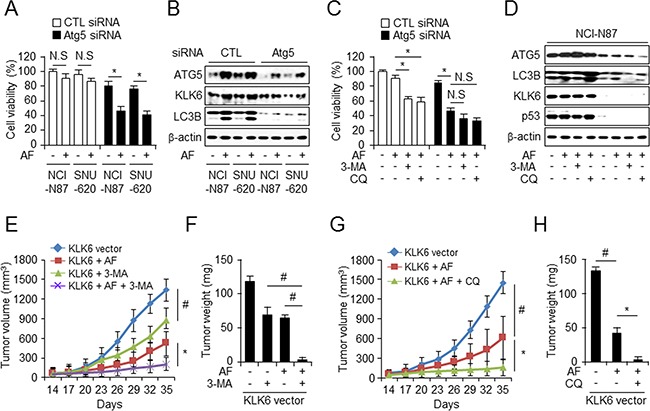
CQ enhances AF-induced growth inhibition of tumor xenografts in KLK6-overexpressing cells WST-1 cell viability assay **A**. and western blots **B**. with ATG5, LC3B, and KLK6 antibodies performed after transfection of ATG5 siRNA or control siRNA into NCI-N87 and SNU-620 cells. WST-1 assay **C**. and western blot analysis **D**. after transfection of ATG5-targeted siRNA into NCI-N87 cells treated with AF and/or 3-MA (5 mM) or CQ (20 μM). Combined treatment with AF (4 mg/kg) in the presence or absence with 3-MA (10 mg/kg) **E**. or CQ (60 mg/kg) **G**. (i.p) injection every other day for the indicated period inhibits tumor volume and tumor weight in the presence or absence of 3-MA **F**. and CQ **H**. of KLK6-overexpressing human gastric cancer xenografts in nude mice (n = 6).

To confirm the involvement of autophagic cell death in acquired resistance to chemotherapy under AF treatment, we inhibited autophagy using siRNA-mediated ATG5 knockdown in NCI-N87 and SNU-620 cells resulted in increased cell mortality statistically after AF treatment (Figure 7A) and verified by western blot analysis of ATG5, LC3B, p53, and KLK6 (Figure 7B). After *ATG5* silencing in KLK6-positive NCI-N87 cells, we observed increased cell mortality and attenuated autophagy upon AF treatment in the presence of autophagy inhibitors (Figure 7C and 7D). Interestingly, 3-MA and CQ treatment decreased ATG5 and LC3B activation and also decreased KLK6 and p53 protein levels and thus had a chemosensitizing effect. These results supported the notion that KLK6-induced autophagy activation may involve AF-induced cell death mechanism. While the tumorigenic properties of KLK6 are well documented, its function through autophagy regulation in chemoresistance has not been studied so far. Therefore, we assessed the effect of autophagy inhibition of xenograft tumor growth *in vivo*. First, we injected AGS cells (1 × 10^7^ cells) stably overexpressing KLK6 in nude mice and divided these into 3 groups that were treated with DMSO, 3-MA, AF alone, and AF plus 3-MA (Figure 7E and 7F) or DMSO, AF alone, and AF plus CQ (Figure 7G and 7H). Analysis of the tumor volumes and weights in these groups indicated that the groups treated with AF alone and with AF plus 3-MA or AF plus CQ had smaller volumes and weights than DMSO-treated group. Notably, the chemotherapy resistance of KLK6-overexpressing cells was inhibited by combined AF plus 3-MA or AF plus CQ treatment. These findings suggested that the resistance to AF treatment is mediated by induction of autophagy in KLK6-overexpressing cells and can be prevented by autophagy inhibitor. This implies that KLK6 provides cells with chemoresistance that is mediated by increased autophagy.

## DISCUSSION

KLK6 expression is increased in many types of tumors and this elevation is related with more aggressive symptoms and poor prognosis [28, 40, 41]; however, the role of KLK6 in the relationship between autophagy regulation and chemotherapy resistance is currently not well understood. Here, we describe a role of KLK6 expression in modulating autophagy, a conserved mechanism that delivers intracellular proteins such as p53 and organelles to the nucleus and autophagosomes. Recent studies showed that KLK6 upregulation is a strong marker of advanced tumor stage [42]. Analysis of 59 human gastric tumors showed KLK6 upregulation at stages III and IV, but low-level expression at stages I and II. We also reported a viability screen of gastric cancer cells using various anti-cancer drugs indicating that AF resistance-related cell death depend on the KLK6 expression level. With the goal of potential repurposing AF for gastric cancer treatment, we demonstrated that AF could induce apoptosis, autophagy-induced cell death, and the autophagy level in the gastric cancer cells. Recently, Rebecca S et al. group reported that Caveolin-1-mediated expression and secretion of kallikrein 6 in colon cancer cells [43]. Caveolae may play an important role in protease secretion and modulate mitogenic signaling as well as protein trafficking, both of which are relevant to KLK6 regulation. It may be an important role for Cav-1 dependent increase in secretion applies to another protease, KLK6, as well. Moreover, another recent report showed that the B1R agonist acts as a functional stimulus for the secretion of KLK1 and KLK6, an event relevant for kinin production and cell invasion, respectively [44]. B1R may have a potential target receptor for KLK6 secretion in cancer.

The results of the current study imply that autophagy induction by KLK6 contributes to the tumorigenesis and chemoresistance in gastric cancer. This emerged from *in vitro* as well as *in vivo* experiments. We observed increased, stage-dependent autophagy upon KLK6 overexpression in gastric cancer tissues and cells treated with AF. Further, overexpression of KLK6 in a xenograft model induced AF-induced chemoresistance, autophagosome formation, and the synthesis and activation of LC3B in mouse gastric tumor tissue. These observations demonstrated that KLK6 expression induces the process of autophagy as a general response to AF treatment in gastric cancer. In the xenograft model using KLK6-overexpressing cells treated with AF alone or with AF plus CQ, we observed that the chemoresistance of KLK6-overexpressing cells was inhibited by combining AF treatment with CQ, indicating that inhibition of autophagy attenuates AF-induced chemoresistance via increased KLK6 levels. Furthermore, knockdown of LC3B enhanced the cell mortality in AF-induced KLK6-overexpressing cells. Conversely, overexpression of LC3B inhibited the mortality in cells with low basal KLK6 expression. These experiments suggested that active LC3B in gastric cancer cells may regulate the chemotherapy resistance that drive anti-tumor drug. In addition, loss- and gain-of-function studies of KLK6 and p53 showed that KLK6 and nuclear p53 were involved in the regulation of autophagy.

Recent studies reported that autophagy is related to resistance to cancer chemotherapy, major therapeutic molecules, and various drugs used for autophagy regulation in cancer therapy. Additionally, autophagy mediates nonapoptotic pathways through a protective (oncogenic) or destructive (tumor-suppressive) role [45]. For example, beclin-1-transfected breast cancer cells have lower sensitivity to the proliferative effects of the estrogen receptor agonist E2 and the growth-suppressive effects of tamoxifen, contributing to acquisition of resistance with a concomitant increase in autophagy [46]. Autophagy can regulate the fate of cancer cells possessing oncogenes (Akt, Ras, and Raf) or tumor suppressor genes (Beclin-1 and p53) during tumor progression [47].

Our results suggested that AF-induced autophagic cell death in gastric tumors contributes to drug resistance and cell survival in gastric cancer cell lines and patient samples and that KLK6 and p53 status play important roles in the chemotherapeutic mechanism, with clinical implications for gastric cancer. The p53–autophagy interaction is controversial. Although p53 is best known for its pro-apoptotic function in cancer, it also performs a prosurvival function [48, 49] and its action as a positive or negative regulator depends on its activation status [50]. Similarly, the function of autophagy in cancer is complex, involving both oncogenic and tumor-suppressive roles [51]. In this study, KLK6 or p53 knockdown restored chemosensitivity and enhanced AF-induced autophagic cell death in gastric cancer cells, whereas KLK6 or p53 upregulation was associated with clinical chemotherapy resistance, autophagic defense mechanism, and tumorigenesis.

To determine whether *KLK6* expression is regulated by transcription factors in gastric tumors during the autophagic process, we performed ChIP analysis of the *KLK6* promoter. Surprisingly, we found that nuclear p53 binds to the promoter and that overexpression of KLK6 and p53 activates an autophagy pathway, in contrast to inhibition of KLK6 and p53. Importantly, p53 localization is a determining factor in the autophagic process [52], where it plays diverse roles. Nuclear p53 acts as an autophagy-inducing transcription factor, while cytoplasmic p53 has an autophagy-inhibitory function [53]. Our microscopic analysis of gastric cancer cells revealed that most cells exhibited nuclear p53 and activation of KLK6 as a pro-autophagic transcription factor, whereas AF-sensitive cells showed increasing amounts of cytoplasmic p53 over time, suppression of autophagy, and cell death. When we regulated the autophagy level using siRNA and overexpressed LC3B as an autophagy marker, KLK6 and p53 were both found to be important for AF-induced autophagic cell death. Expression of both KLK6 and p53 was regulated by inhibition of autophagic flux and, in particular, co-immunoprecipitation assay demonstrated a strong KLK6-LC3B interaction.

Recent studies have reported a role of KLK6 in the regulation of membrane-dependent signaling processes [54], and in neurodegenerative diseases such as Alzheimer's disease and Parkinson's disease [55]. KLK6, a trypsin-like serine protease, is localized to the endoplasmic reticulum (ER) and then secreted [56]. Various lines of evidence have indicated that membranes from the ER, mitochondria, Golgi, and endosomes, and the plasma membrane could all potentially act as a source of autophagosomal membrane in non-specialized macroautophagy [57]. Recent investigations have refined the localization of autophagosome formation to ER–mitochondria contact sites [57]. We demonstrated that KLK6 regulates the autophagosome localization of autophagic protein LC3B and nucleus localization of transcription factor p53, here reported to regulate KLK6 transcription. The occurrence of binding components such as Egr-1 [58] transcription factor such as Egr-1, membrane protein as caveolin-1 [58], and DNA binding protein as HMGB1 [59] in autophagosomes and their associations with LC3B under basal states imply the regulatory role of KLK6 in the activation of autophagy. Our observation that KLK6 overexpression mobilizes these components from the plasma membrane and cytosol to the nucleus, in association with autophagic activation, suggests that these translocations represent early events in the activation of autophagy after AF treatment. Further, our data indicated time-dependent dissociation of LC3B or p53 from KLK6 in the autophagosomes or nucleus in response to AF treatment. These results suggest an upstream regulatory switch for activation of autophagy with AF treatment in gastric cancer cells. Our study provides new insight into the repositioning of anti-rheumatoid arthritis drugs for gastric cancer treatment. KLK6 expression increases AF-induced tumor survival activity in gastric cancer and autophagy induction via p53 activation. The association of *KLK6* gene with increased autophagic activity was exploited towards establishing novel as well as efficient chemotherapy resistance treatment protocols, which involve either inhibitors of autophagy as a single agent treatment or their rational combination with AF.

In conclusion, *KLK6* may be an autophagy-related and p53-dependent gene in several tumor microenvironments. Modulation of the KLK6 status regulating AF-induced autophagic cell death is a potential therapeutic strategy for gastric cancer. Combined treatment with AF and inhibitors of autophagy such as CQ may yield even higher treatment efficacy in cases that more strictly depend on autophagy for the regulation of tumor growth. Changes in transcription factor binding or interactions both inside and outside the nucleus might determine which of the dual roles of autophagy is elicited, based on the status of KLK6 and p53. We anticipate the development of novel chemotherapeutic strategies based on AF-induced autophagic cell death.

## EXPERIMENTAL PROCEDURES

### Cell culture

The Stomach cancer cell lines AGS, SNU-216, NCI-N87, SNU-620, SNU-638, SNU-668, and NUGC-3 were purchased from the Korean Cell Line Bank (Cancer Research Center, Seoul National University, Seoul, Korea)and grown in RPMI1640 medium (Gibco) supplemented with 5% fetal bovine serum (Gibco) and 100 μg/ml antibiotics (100 U/ml penicillin and 100 μg/ml streptomyhcin, Gibco). Cells (1x10^5^ cells/well) were plated in 24-well cell culture plates and grown at 37°C in a humidified, 5% CO_2_/air atmosphere. Cell viability assayed by WST-1. Please see other experimental procedures in Supplemental Experimental Procedures.

### Cell viability assay

WST-1 assay was performed according to the manufacturer's instructions (Roche, Mannheim) with 10 μl of WST-1 reagent was added to each well of a 96-well plate (1 × 10^3^ cell/well). After 1h of incubation using CO_2_ incubator, the conversion of WST-1 reagent into chromogenic formazan was monitored with a spectrophotometer. On day 1 after plating, cells were treated with various doses (1, 2.5, 5 and 10 μM) and times (4, 8, and 16 hrs) of AF (Enzo, NY). A methyltransferase inhibitor, 5’-Aza-2’-deoxycytidine (Sigma, St. Louis MO), was added to the culture medium 1 μM for 72 hr to induce demethylation of the cytosine residues, respectively.

### Transient and stable transfection

AGS, SNU-216, NCI-N87 and SNU-620 cells (1 × 10^5^ cell/well) in 24-well plate were transfected with these double-stranded siRNAs (30 nmol/ml) such as siKLK6, siLC3B, and sip53 (Bioneer) for 24 hr by the Lipofectamine 2000 (Invitrogen) method according to the manufacturer's protocol and recovered in RPMI1640 medium (Welgene) containing 10% fetal bovine serum for 24 hrs. After recovering, viable cells were calculated by WST-1. pcDNA3.1-KLK6 and pEGFP-LC3B and –KLK6 made to study of target gene and pCMV-Neo-Bam p53 (addgene) purchased. KLK6 stable overexpressed cell line was constructed by using pcDNA 3.1 – KLK6 plasmid. In stable overexpressed cell lines, AGS cell (1x10^5^ cell/well) was seeded into 24-well plate. After 16 hr, KLK6-expressed vector was transfected into AGS cells using lipofectamine 2000 (Invitrogen). The following day, the medium was changed, and G418 was added to the culture medium to a final concentration of 800 μg/mL and cultured in the presence of G418 for 4 weeks. Medium was exchanged every 3 day. The expression of KLK6 to identify establishment of KLK6 stable cell line was checked by RT-PCR and Western blot assay.

### Isolation of genomic DNA, total RNA and protein

Chromosomal DNA from gastric cells (2 × 10^6^ cells/well) plated in 100 mm cell culture plates and tissues (approximately 50–100 mg) were extracted using a genomic DNA purification kit (Promega, Madison, WI) according to the manufacturer's protocol. The extracted DNA was eluted with 250 μl of distilled water. Total RNA from gastric cells (2 × 10^6^ cells/well) in 100mm cell culture dish and tissues (approximately 50–100 mg) was prepared using Trizol according to the manufacturer's protocols (invitrogen, Carlsbad). Genomic DNA and total RNA from cultured cells and tissues were prepared using an All Prep DNA/RNA Mini kit (Qiagen, Valencia) with elution of 100 and 30 μl, respectively. Protein cell lysates were collected in RIPA buffer containing a protease inhibitor cocktail (Sigma) on ice for 30 min and passed through an 18-gauge needle, and spin down. Supernatant was analyzed for protein content using the BCA method (Thermo scientific, Pierce BCA Protein Assay Kit).

### Methylation-specific polymerase chain reaction (MSP)

Sodium bisulfite modification of genomic DNA was carried out using an EpiTect Bisulfite kit (Qiagen) according to the manufacturer's protocol using 0.1 mg of purified DNA. Briefly, primer sequences were designed using the Methprimer program ( http://www.urogene.org/methprimer/index1.html). Quantitative PCR was performed using a Power SYBR Green Kit (Applied Biosystems, Foster City) according to the manufacturer's protocol. A methylation index was calculated for each sample using the following formula: methylation index = 1 / [1 + 2^−(CTu − CTme)^] × 100%, where CTu is the average cycle threshold (CT) obtained from duplicate quantitative PCR analyses using the unmethylated primer pair, and CTme (Supplementary Table S1) is the average CT obtained using the methylated primer pair.

### qPCR and western analysis

KLK6 and LC3B expression levels were measured by qPCR analysis using cDNA synthesized from 5 μg of total RNA and a reverse transcription kit (Promega, Madison, WI). One microliter of cDNA was used for the PCR, and duplicate reactions were performed for each sample using a ABI Power SYBR green PCR Master Mix (Applied Biosystems, Warrington) with gene-specific primers (Supplementary Table S1) on an ABI Step one plus instrument (Applied Biosystems). RNA quantity was normalized to GAPDH content, and gene expression was quantified according to the 2^-ΔCt^ method.

In Western blot analysis, to conduct western blotting, cancer cell lines were solubilized in radioimmunoprecipitation assay (RIPA) lysis buffer (50 mM/L Tris-HCl (pH 7.4), 150 mM/L NaCl, 1% NP40, 0.25% sodium deoxycholate, 1 mM/L phenylmethylsulfonylfluoride (PMSF), 1 mM/L sodium orthovanadate, 1x sigma protease inhibitor cocktail) and protein was measured using a standard bicinchoninic acid assay. Equal amounts of protein (20-50 μg) were size-fractionated by 10~15% SDS-PAGE and then transferred onto PVDF membrane (Millipore Corporation, Billerica, MA, USA). Membranes were blocked by incubation for 1 hr with 5% skim milk/PBS-T buffer (PBS with 5% powdered milk and 1% Triton X-100), and incubated overnight at 4°C with primary antibodies diluted in 1 × PBST buffer. The following primary antibodies were used: β-actin, KLK6, p53, MDM2, Atg5, LAMP-1, LAMP-2, LAMP-3 (SantaCruz, 1:1000), LC3B, Beclin-1 (Sigma, 1:1000), cleaved caspase-3, -9, PARP, Akt, p-Akt, p38, p-p38 and JNK (CellSignaling, 1:700). The membranes were washed 3 times with PBST. Secondary antibodys were diluted in PBST and were added for 40 minutes at room temperature. The following secondary antibodies were used: anti-rabbit IgG HRP-linked antibody and anti-mouse IgG HRP-linked antibody (Sigma, 1:6000). The membranes were washed 6 times with PBST for 1 hour. The blots were visualized by chemiluminescence (Clarity Western ECL; Bio-rad).

### Chromatin immunoprecipitation-qPCR (ChIP-qPCR)

ChIP assays were performed using an EZ ChIP Chromatin Immunoprecipitation kit (Millipore, Billerica, MA, USA) as described in the supplier's protocol. AGS, SNU-216, NCI-N87 and SNU-620 cells (3 × 10^6^ cells/well) were seeded in 100 mm cell culture plate and cultured for 24 hour. After washing with 1x PBS, added 140 μl of 37% Formaldehyde and incubated at room temperature for 15 minute. Following to added 740 μl Glycine (1 M), cells collected with cell scraper (SPL) after washing with PBS. After centrifuged, added 1 ml of SDS lysis buffer. Briefly, the cross-linked chromatin was sonicated after cell lysis and then incubated with antibodies against p53 (SantaCruz) at 4°C overnight. The lysates were precipitated with Protein A-agarose (Millipore), and the beads were washed, sequentially treated with 10 μl of RNase A (37°C for 30 min) and 75 μl of Proteinase K (45°C for 4 h), and incubated at 65°C overnight to reverse cross-link the chromatin. The DNA was recovered by phenol-chloroform extraction and coprecipitation with glycogen, and dissolved in 50 μl of Tris-EDTA (TE) buffer. DNA associated with the p53 was amplified by PCR using 1 μl of the precipitated DNA. PCR primers (Supplementary Table S2) were designed to amplify the p53 binding sequence at the promoter of KLK6. The qPCR conditions were 40 cycles at 94°C for 40s, 60°C for 1 60s, and 72°C for 40s.

### Immunoprecipitation (IP)

We extracted cell lysates from AGS, SNU-216, NCI-N87 and SNU-620 cells (2 × 10^6^/well) on 100 mm cell culture plate in buffer containing 50 mM Tris-HCl, pH 7.5, 250 mM NaCl, 5 mM EDTA, 0.5% (v/v) NP-40 and protease inhibitor cocktail (Sigma). We incubated anti-LC3B (Sigma), anti–KLK6 (Santa Cruz) and anti-p53 (Santa Cruz) with lysate at 4°C for 16 hrs. We used protein A/G PLUS agarose (Santa Cruz) to pull down immunocomplexes. We washed precipitates three times with 50 mM Tris-HCl, pH 7.5, 250 mM NaCl, 5 mM EDTA, and 0.5% (v/v) NP-40. We resolved the immunoprecipitated proteins by 15% SDS-PAGE and analyzed them by immunoblot.

### Immunofluorescent (IF)

After AF treatment, AGS and NCI-N87 were fixed for 15 min with 4% paraformaldehyde and stained by cell staining for immunofluorescence microscopy method according to the manufacturer's protocol (Life technology). The slides were incubated with blocking buffer (1% BSA in PBS) for 30 min at 37°C and exposed overnight to anti-LC3B (Sigma), anti-KLK6 (Santa Cruz) and anti-p53 (Santa Cruz) antibody (1:200 dilution in blocking buffer) at 4°C. Secondary antibody was stained using red-fluorescent Alexa Fluor 555 goat anti-rabbit and -mouse, and green-fluorescent Alexa Fluor 488 goat anti-rabbit and -mouse. Nuclear DNA was stained with DAPI (Sigma). Samples were analyzed with a ZEISS LSM 510 META confocal microscope.

### DAPI staining assay

Morphological changes of the nuclear in AGS, SNU-216, NCI-N87 and SNU-620 cells (1 × 10^5^/well) plated 6-well plate undergoing apoptosis were detected by staining with DAPI (Sigma). Gastric cancer cells were grown on glass coverslips and treated with the presence or absence of AF for 8 hr. Cells were washed twice with PBS and fixed by incubation in 4% paraformaldehyde for 30 min. Following washing with PBS, cells were incubated in a DAPI solution (1 μg/ml) for 30 min in the dark. Cells were then washed with PBS and subjected to fluorescence microscopy.

### Quantification of pEGFP-LC3 puncta

AGS, SNU-216, NCI-N87 and SNU-620 cells (1x10^5^ cell/well) were transfected with pEGFP-LC3B and control pEGFP-N2 using lipofectamine 2000 (Invitrogen), and then treated with AF 2.5 μM for 8 hrs. pEGFP-LC3B-positive punctate pattern was observed by confocal microscopy. Confocal microscopy was conducted using a ZEISS LSM 510 META confocal microscope with 405- and 488-nm excitation lasers.

### Immunohistochemistry (IHC) using human gastric normal/cancer tissue samples

Tissue microarray slides for human gastric normal and tumor were purchased from SuperBioChips (SuperBioChips Laboratories, Seoul). Tissue sections were deparaffinized by xylene and blocked by normal serum. Primary antibody was diluted with anti-KLK6 (Santa Cruz Biotechnology, Inc.), anti-LC3B (Sigma) and anti-p53 (Santa Cruz Biotechnology, Inc.) antibody with CAS blocking solution (Invitrogen) for overnight. And slides incubated with biotinylated secondary antibody for overnight. Using DAB substrate kit (DAB substrate kit, vector laboratories), tissue slides stained with substrate at room temperature until suitable staining develops immunohistochemistry staining was performed as the manufacturer recommended (VECTASTAIN ABC KIT, Vector laboratories).

### Flow cytometry (FACs)

To detection apoptosis, cells were stained as described in the FITC Annexin V Apoptosis Detection Kit (BD Pharmingen, San diego) and counted by flow cytometry (BD FACs Calibur). FITC Annexin V Apoptosis Detection was perfomed as the manufacturer recommended (BD Pharmingen).

### Luciferase reporter assay

The promoter region (-0.1 - to -1 kb) of KLK6 was amplified by PCR primers (Supplementary Table S3) from human genomic DNA and cloned into the Mlu I (New England Biolabs) and Xho I (New England Biolabs) sites of with pGL3 luciferase vector (Promega). The PCR (ABI2720) was carried out with 35 cycles at 94°C for 40 sec, 56°C for 40 sec, then 72°C for 1 minute. One day before transfection, AGS and NCI-N87 cells (2 × 10^5^ cell/well) were seeded in 6-well plate and transfected with 2.5 μg of pGL3 luciferase vector (Promega) after 24 hr using lipofectamine 2000 (Invitrogen). Luciferase activity was measured 36 hours after transfection in two independent cultures using a dual-luciferase reporter assay kit (promega) on Molecular Devices Filter Max F3 (Sunnyvale).

### LDH assay

AGS, SNU-216, NCI-N87 and SNU-620 cells (1 × 10^4^ cells/well) was seeded into 96-well plate with growth medium. To determine the LDH (Roche) activity in supernatants, 100 μl of Reaction mixture added and incubated for 30 minutes in dark room. LDH activity measured absorbance of the samples at 490 or 492 nm using ELISA reader.

### Cytoplasmic and nuclear fractionation

Fractionation of cytoplasmic and nuclear extracts was carried out using Nuclear Extract kit (Active motif) according to manufacturer's instructions. Supernatants used as cytoplasmic fraction, whereas pellets were resuspended in 50 μl of complete lysis buffer and supernatants were used as nuclear fractions by centrifuging at 14,000 × *g* for 10 minutes at 4°C

### Xenograft assay by AF in nude mice

Female 6- to 8-week-old female athymic nu/nu nude mice were purchased from Central Lab, Animal Inc (Seoul) and used for the *in vivo* experiments. Mice were housed in a pathogen-free barrier room in Animal Care Facility at the Korea Research Institute of Bioscience and Biotechnology (KRIBB). This experimental protocol was reviewed and approved by the Institutional Animal Care and Use Committee of the Korea Research Institute of Bioscience and Biotechnology (KRIBB) and performed in accordance with the guide for the Care and Use of Laboratory Animal published by the U.S. National Institutes of Health (NIH Publication, 8^th^ Edition, 2011). Two clones of AGS cells stably expressing pcDNA3.1-KLK6 and control pcDNA3.1-transfected cells were used in a xenograft assay. The mice were divided into 4 groups, with 8 mice per group: (1) AGS pcDNA3.1 stable expressing cell, (2) AGS pcDNA3.1 stable expressing cell and AF (4 mg/kg), (3) AGS pcDNA3.1-KLK6 stable expression cell, and (4) AGS pcDNA3.1-KLK6 and AF (4 mg/kg). AF was dissolved in PBS and mice were given AF intraperitoneally (i.p.) every other day (weekends off). For the xenograft assay, cells were collected by centrifugation, washed twice in phosphate-buffered saline (PBS), 1 × 10^7^ AGS cells were resuspended in 0.1 ml of PBS, and injected s.c. into right dorsal flank of nude mice (eight mice per cell line) using 25-gauge needles. When tumor volumes monitored approximately 200 mm^3^, treatment with AF were started and were monitored twice weekly. Moreover, the tumor volumes of mice were measured with calipers and calculated using the following formula: (A × B^2^)/2, where A is the largest and B is the smallest diameter.

### Statistical analysis

All results were confirmed in at least three independent experiments; data from one representative experiment are shown. All quantitative data are presented as mean±standard deviation (SD); *In vivo* data are expressed as mean±standard error of the mean (SEM). Statistical analysis was performed using SAS 9.2 software (SAS Institute, Cary). Student's *t*-tests were used for comparisons of means of quantitative data between groups and *p*<0.05 was considered statistically significant.

## SUPPLEMENTARY MATERIALS FIGURES AND TABLES


