# Supplementary material for: Kallikrein-related peptidase 6 induces chemotherapeutic resistance by attenuating auranofin-induced cell death through activation of autophagy in gastric cancer
Source: Oncotarget. 2016 Nov 15;7(51):85332–48. doi: 10.18632/oncotarget.13352 (PMC5356740; doi:10.18632/oncotarget.13352)
Supplement: Supplementary file 1 [file oncotarget-07-85332-s001.pdf]

Kallikrein-related peptidase 6 induces chemotherapeutic resistance by attenuating auranofin-induced cell death through activation of autophagy in gastric cancer

SUPPLEMENTARY FIGURES

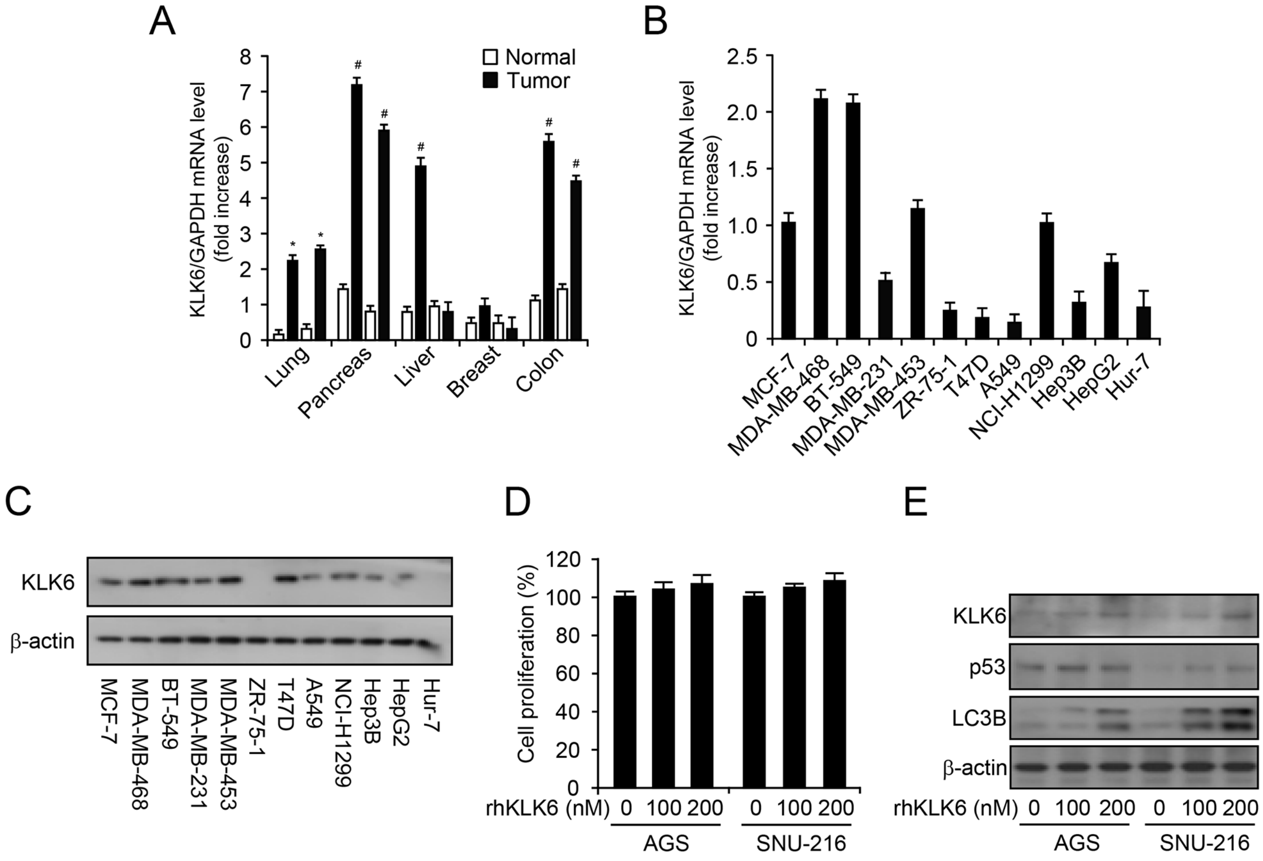

Supplementary Figure S1: Differential KLK6 expression in various tissues and cancer cell lines. A-B. qPCR is performed to various tissues such as lung, pancreas, liver, breast and colon and various cancer cell lines including MCF-7, MDA-MB-468, BT-549, MDA-MB-231, MDA-MB-453, ZR-75-1, T47D, A549, NCI-H1299, Hep3B, HepG2 and Hur-7 cells to identify KLK6 mRNA level. **C.** Western blots showing using anti-KLK6 antibody on MCF-7, MDA-MB-468, BT-549, MDA-MB-231, MDA-MB-453, ZR-75-1, T47D, A549, NCI-H1299, Hep3B, HepG2 and Hur-7 cells. **D.** Recombinant KLK6 protein measured by WST-1 and gastric cancer cell line, AGS and SNU-216 were grown for 72 hr in growth media containing0, 100 and 200 nM. Bars are means (± SD) value of 3 reactions. **E.** Western blots showing reactions of anti-KLK6, -p53 and -LC3B antibodies in AGS and SNU-216 cells with treated recombinant KLK6 proteins including 100 and 200 nM.

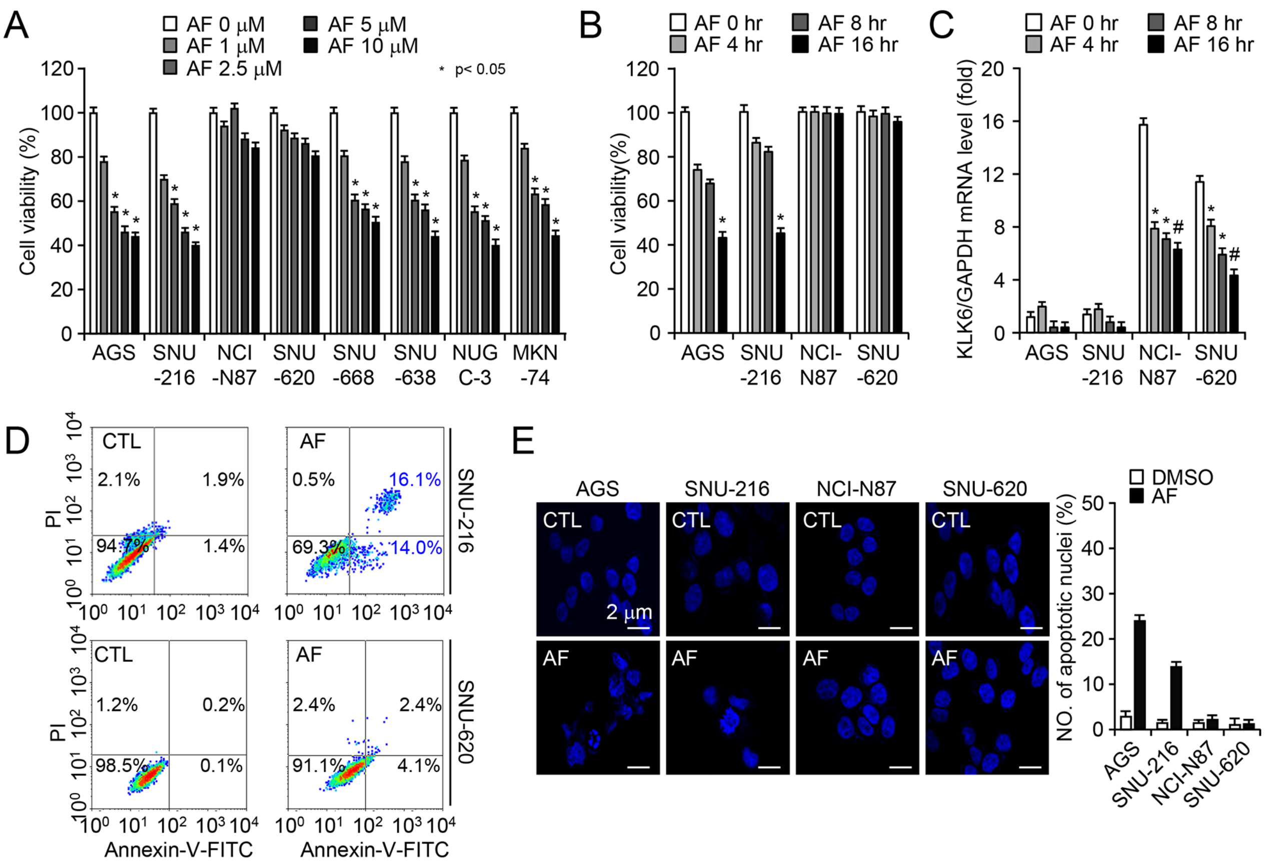

Supplementary Figure S2: The level of KLK6 expression is associated with AF-induced cell death on gastric cancer cell lines. A. Cell viability assay by WST-1 was fulfilled on diverse concentration with AF 1, 2.5, 5 and 10 μM in gastric cancer cell lines including AGS, SNU-216, NCI-N87, SNU-620, SNU-638, SNU-668, NUGC-3 and MKN-74 cells. (*$p<0.05$). **B.** Cell viability assay by WST-1 was performed on various times such as 4, 8 and 16 hr of AF 2.5 μM in AF-sensitive gastric cancer cell lines including AGS and SNU-216, and in AF-resistant gastric cancer cell lines including NCI-N87 and SNU-620 (*$p<0.05$). **C.** qPCR showing KLK6 mRNA levels on AF-time dependent manner in AGS, SNU-216, NCI-N87 and SNU-620 cells. **D.** Besides Figure 2B, annexin-V assay for apoptosis detection using FACs also is performed in SNU-216 and SNU-620 cells. **E.** Apoptosis was detected by DAPI staining in AF-induced gastric cancer cell lines and determined the proportion of apoptotic cells. 400x. Scale bars = 2 μm. images of DAPI staining with triple reactions analyzed on random 100 cells of DAPI positive staining cells and number of AF-induced apoptotic nuclei cells also calculated.

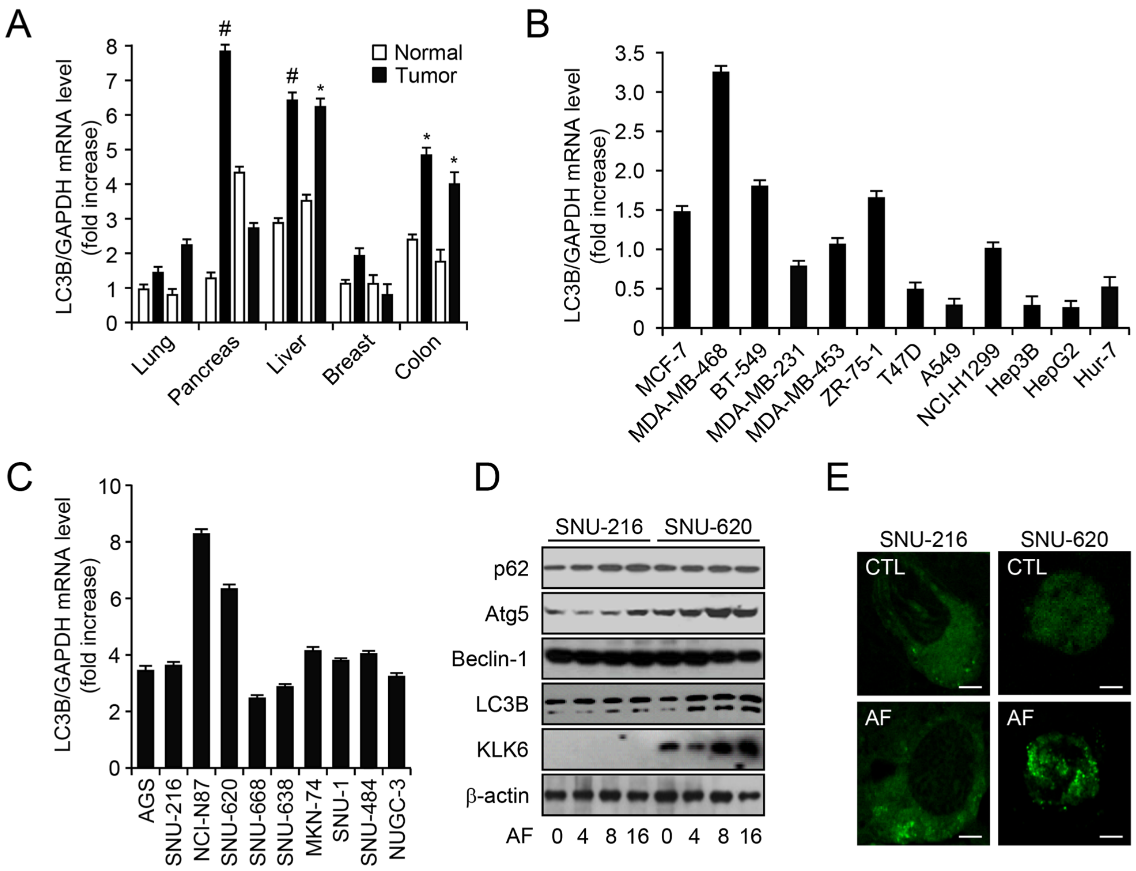

Supplementary Figure S3: Differential LC3B expression in various tissues and cancer cell lines. A-B. With same sample of Supplementary Figure S1A-B, qPCR is performed to various tissues such as lung, pancreas, liver, breast and colon and various cancer cell lines including MCF-7, MDA-MB-468, BT-549, MDA-MB-231, MDA-MB-453, ZR-75-1, T47D, A549, NCI-H1299, Hep3B, HepG2 and Hur-7 cells to identify LC3B mRNA level (*$p<0.05$, # $p<0.01$). **C.** qPCR showing LC3B mRNA levels in gastric cancer cell lines including AGS, SNU-216, NCI-N87, SNU-620, SNU-638, SNU-668, NUGC-3 and MKN-74 cells. **D.** With Figure 3B data, Western blots analyses showing differential expression of autophagy markers such as Atg5, Becn-1 and LC3B with KLK6 expression level in AF-time dependent manner on SNU-216 and SNU-620 cells. **E.** Similar to Figure 3C results, EGFP-LC3 vector was placed under AF treatment for 16hr on SNU-216 and SNU-620 cells compare with treated DMSO and indicator of autophagy activation, dots was more observed in AF-sensitive SNU-216 cells. (*Continued*)

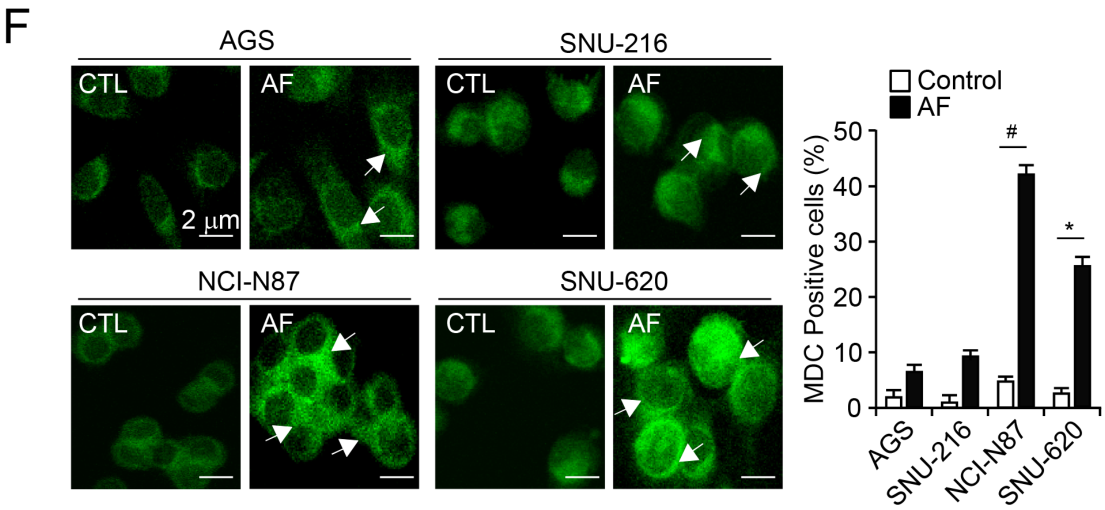

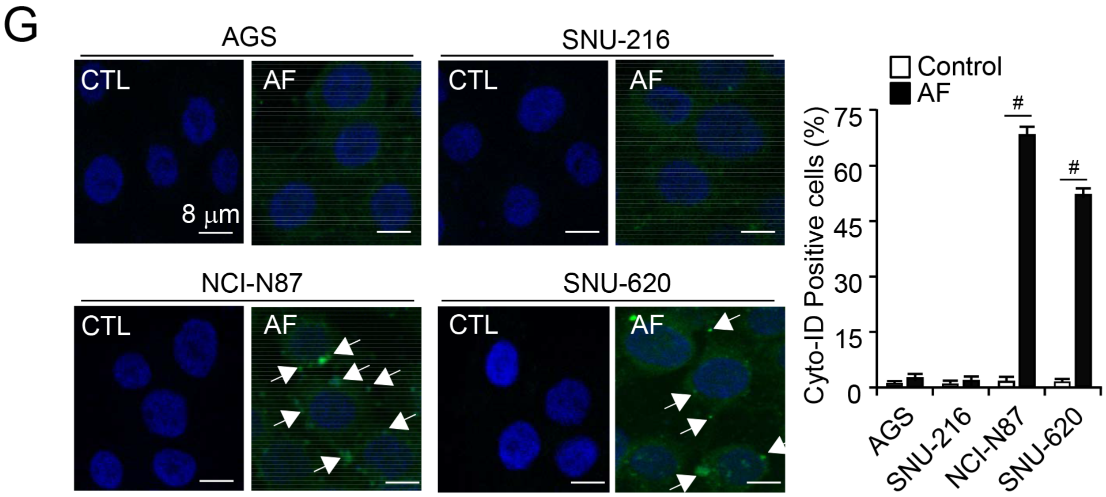

Supplementary Figure S3: (*Continued*) Differential LC3B expression in various tissues and cancer cell lines. F-G. MDC and Cyto-ID staining were used for detection of autophagy activation in AF-induced gastric cancer cells. Images of MDC and Cyto-ID staining with triple reactions analyzed on random 100 cells of MDC and Cyto-ID positive staining cells and number of punctated cells also calculated; 400x plus zoom, Scale bars= 2 µm and 8 µm, respectivelly (*p<0.05, # p<0.01).

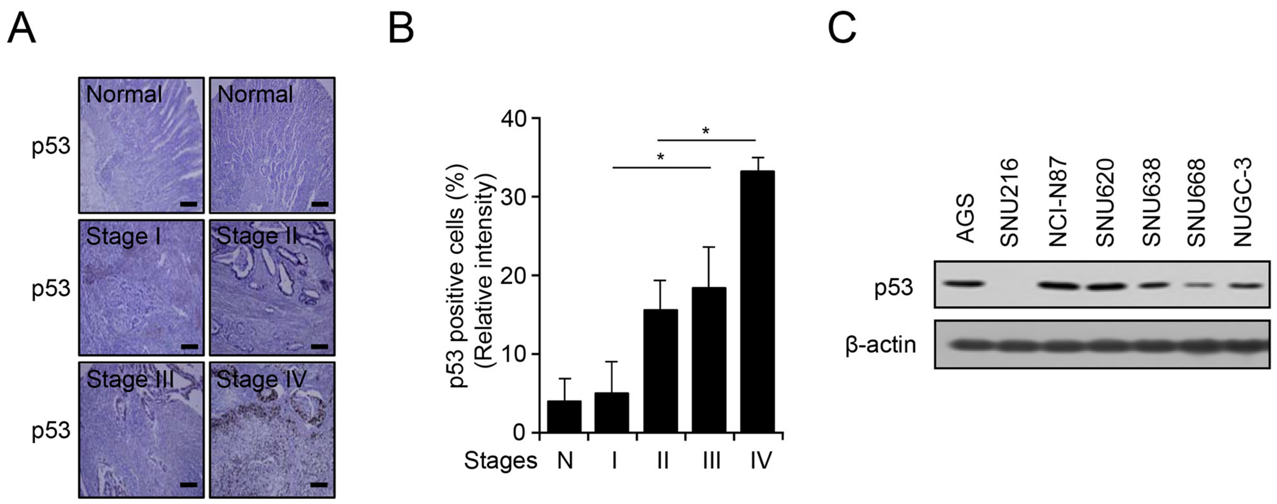

Supplementary Figure S4: p53 expression levels in gastric cancer cell lines and tissues. A. Immunohistochemistry (IHC) showing reactions against anti-p53 antibody in 59 paired-normal and cancer tissues including 18 sample of tumor stage I, 14 sample of tumor stage II, 24 sample of tumor stage III and 3 sample of tumor stage IV of gastric cancer patients. Original magnification, 200x, Scale bars, 50 µm. **B.** Histogram shows intensity value of p53-positive cells depend on tumor stages (*p<0.05). **C.** Western blotting showing using anti-p53 antibody on AGS, SNU-216, NCI-N87, SNU-620, SNU-638, SNU-668 and NUGC-3 cells.

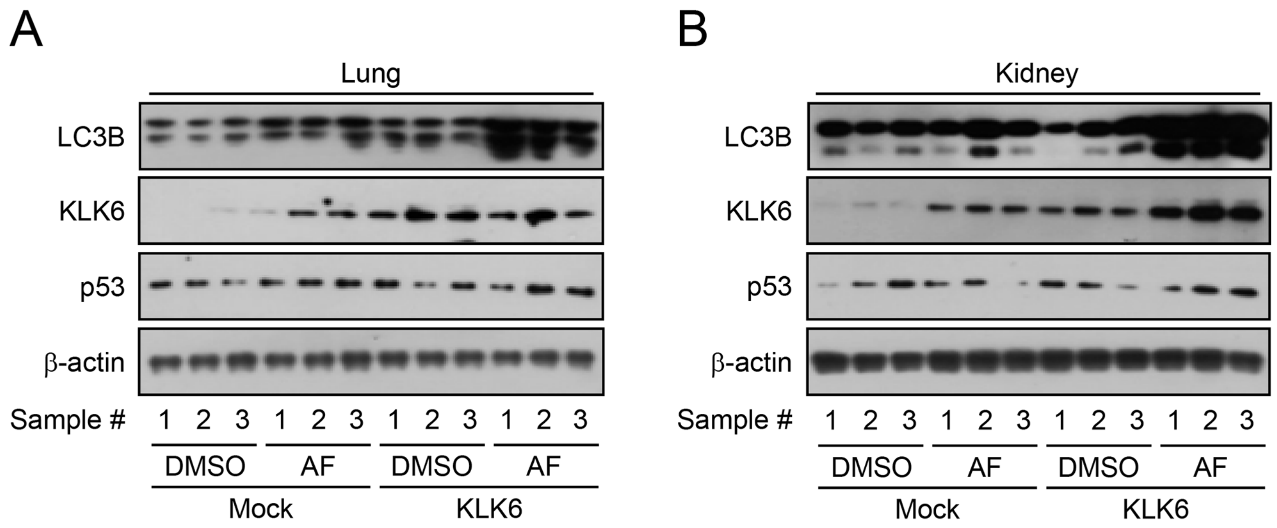

Supplementary Figure S5: Western blots analysis on lung and kidney from 4 groups of AF-treated xenograft mice. In addition to Figure 7D, **A-B.** Western blots analysis of lung and kidney produced by 4 groups including Mock vector, Mock vector plus AF, KLK6 overexpression vector and KLK6 overexpression vector plus AF showing results by anti-LC3B, -KLK6 and -p53 antibodies.

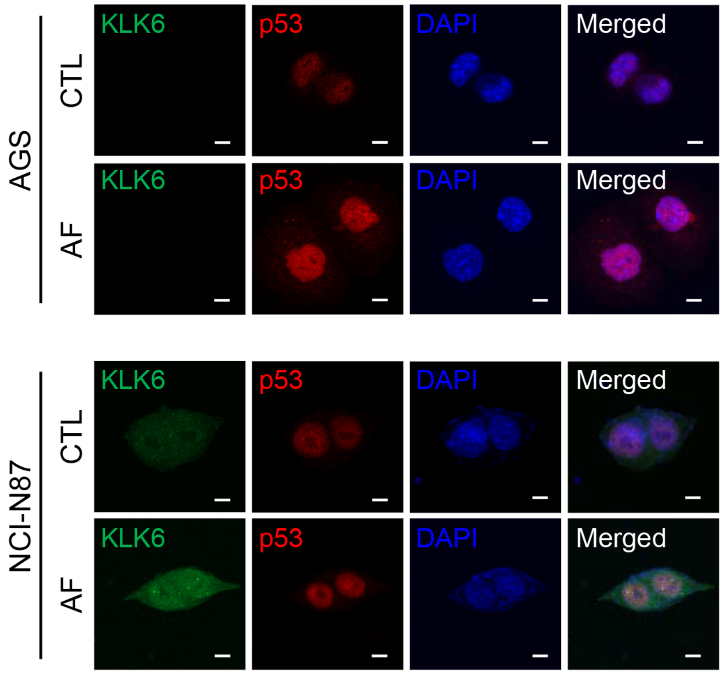

Supplementary Figure S6: Protein interaction between KLK6 and p53 is important to KLK6-induced autophagy activation. Immunofluorescence showing reactions against anti-KLK6 and –p53 antibodies with DAPI staining in AGS and NCI-N87 cells, respectively and expression and localization in presence and absence of AF treatment, and merged images of DAPI, KLK6 and p53; 800x plus zoom, Scare bar = 20 µm.

Supplementary Table S1: Primers used in RT-PCR, Real-time PCR and MSP

RT-PCR	KLK6	Forward	5’-GCAAGACAGCAGATGGTG-3’
		Reverse	5’-CACTTGGCCTGAATGGTTTT-3’
	GAPDH	Forward	5’-CATGACCACAGTCCATGCCAT-3’
		Reverse	5’-AAGGCCATGCCACTGAGCTTC-3’
Real-time PCR	KLK6	Forward	5’-GAAGCATAACCTTCGGCAAA-3’
		Reverse	5’-GGGAAATCACCATCTGCTGT-3’
	LC3B	Forward	5’-AGCAGCATCCAACCAAAATC-3’
		Reverse	5’-CTGTGTCCGTTCACCAACAG-3’
MSP	KLK6	Forward-(M)	5’-AAAAGGAAGTTATTGATGTAATCGT-3’
		Reverse-(M)	5’-AAAAACAATCGAACTTTATCCG-3’
		Forward-(U)	5’-AAAAGGAAGTTATTGATGTAATTGT-3’
		Reverse-(U	5’-CCAAAAACAATCAAACTTTATCCA-3’

Supplementary Table S2: Primers used in ChIP

KLK6	Region 1	Forward	5’-GGCAGTCAGAGACTGCAAAGGAGGA-3’
		Reverse	5’-TGGGGAAAGGGCCCAGGAACAATC-3’
	Region 2	Forward	5’-AGCACTTTGGGAGGCCAAGGTG-3’
		Reverse	5’-TGCAACCTCCGCCTCCTGAGTT-3’
	Region 3	Forward	5’-AGCACCACCATCTTATCACAG-3’
		Reverse	5’-GAAGTAGCGGGGATGATTCCCGAG-3’
	Region 4	Forward	5’-AACCACTGCCTGTGGTTTCCCATGT-3’
		Reverse	5’-CTGTGATAAGATGGTGGTGCT-3’
	Region 5	Forward	5’-CACTCCAGCCTAGGCGACAGAACA-3’
		Reverse	5’-GGCAGAGTGGAGAGAAATGAGGAG-3’

Supplementary Table S3: Primers used in Luciferase promoter assay

KLK6	-100 bp	Forward 5’-ACGCGTAGGGACAAAAGGAA-3’
	-300 bp	Forward 5’-ACGCGTCAGAGACTGAGATG-3’
	-500 bp	Forward 5’-ACGCGTACTGCTTGAACTCA-3’
	-700 bp	Forward 5’-ACGCGTAGAGGCCGGGCACA-3’
	-900 bp	Forward 5’-ACGCGTGTGTCCGCATCTCA-3’
	-1000 bp	Forward 5’-ACGCGTCCAGATCCCTCTGC-3’
	-100 bp ~ -1000bp	Reverse 5’-CTCGAGCCCCCAGCACCCCA-3’
